# Investigating the Influence of PbS Quantum Dot-Decorated TiO_2_ Photoanode Thickness on Photoelectrochemical Hydrogen Production Performance

**DOI:** 10.3390/ma17010225

**Published:** 2023-12-31

**Authors:** Yeonjae Kim, Joo-Won Seo, In-Hee Lee, Jae-Yup Kim

**Affiliations:** Department of Chemical Engineering, Dankook University, Yongin 16890, Republic of Korea; yeonjai95@dankook.ac.kr (Y.K.); wndnjs1105@gmail.com (J.-W.S.); lnh7961@dankook.ac.kr (I.-H.L.)

**Keywords:** photoelectrochemical, hydrogen production, PbS, quantum dots, photoanode thickness

## Abstract

To maximize the photoelectrochemical (PEC) hydrogen production performance of quantum dot (QD)-decorated photoelectrodes, it is crucial to prioritize the optimization of electrode’s structure, including thickness and porosity. In this study, we prepare PbS QD-decorated mesoporous TiO_2_ photoanodes for PEC hydrogen production, and systematically investigate the influence of the photoanode thickness on optical properties and PEC performances. As the thickness of photoanodes increases from 6.4 µm to 16.3 µm, the light absorption capability is enhanced across the entire visible and near-infrared (IR) spectrum due to the improved loading of PbS QDs. However, the photocurrent density is optimized for the 11.9 µm thick photoanode (15.19 mA/cm^2^), compared to the 6.4 µm thick (10.80 mA/cm^2^) and 16.3 µm thick photoanodes (11.93 mA/cm^2^). This optimization is attributed to the trade-off between the light absorption capability and the efficient mass transfer of the electrolyte as the photoanode thickness increases, which is confirmed by the lowest charge transfer resistance (*R*_ct_) evaluated from the electrochemical impedance data.

## 1. Introduction

To effectively address the rapidly increasing energy demand and the threat of global warming, the efficient utilization of clean and renewable energy is essential. Research aimed at maximizing the potential of solar energy is considered a key element in sustainable energy supply and climate change mitigation. One of the most promising approaches for the efficient utilization of solar energy and the generation of renewable and eco-friendly energy is photoelectrochemical (PEC) hydrogen production [1,2,3,4,5]. 

As light-absorbing materials utilized in solar energy devices, quantum dots (QDs) have garnered significant attention. QDs exhibit distinct electrical and optical properties because of the quantum confinement effect, differentiating them from bulk materials [6,7]. The unique optical characteristics of QDs, coupled with advantages such as multiple-exciton generation (MEG), high absorption coefficients, and facile bandgap control, have led to substantial interest in the research fields of solar cells and PEC hydrogen pro-duction [8,9,10,11]. 

Moreover, introducing the QDs with a relatively small band-gap (*E*_g_ < 1.5 eV) as photosensitizers allows for the effective utilization of incident light not only in the visible spectrum but even in the near-infrared range. In particular, PbS QDs can be effectively employed in PEC hydrogen production as the light-absorbing material because of their broad absorption spectrum and high quantum efficiency [12,13,14]. Studies have reported the enhancement of the light absorption spectrum and outstanding PEC hydrogen production performance by decorating PbS QDs on wide band-gap semiconductors like TiO_2_ and BiVO_4_ [15,16,17,18].

To maximize the PEC hydrogen production performance of such QD-decorated photoelectrodes, it is crucial to prioritize the optimization of the electrode’s structure, including thickness and porosity. As the thickness of the photoelectrode increases, it can absorb a larger quantity of photons per unit of active area. However, beyond a certain thickness, drawbacks such as increased electron recombination with the redox couple in the electrolyte and slowed mass transfer within the electrolyte may arise for photoelectrodes [19,20,21]. Therefore, it is necessary to investigate the influence of the thickness of QD-decorated photoanodes on the performance of PEC hydrogen production. For instance, in the field of QD-sensitized solar cells, sufficient research has been conducted on the variation and optimization of photovoltaic performances according to the thickness of photoelectrodes [22,23]. However, in the field of PEC hydrogen production, such research endeavors have been scarcely pursued, despite their fundamental necessity [24,25].

In this study, we coated PbS QDs onto the surface of mesoporous TiO_2_ films through successive ionic-layer adsorption and reaction (SILAR) process and optimized the thickness of the photoelectrode to maximize PEC hydrogen production performance. The morphologies and uniformity, as well as the chemical states of the PbS QDs on the TiO_2_ surface, were characterized. In addition, we systematically investigated the impact of the photoelectrode thickness on its optical properties and PEC hydrogen production characteristics. Additionally, we specifically explored the charge transfer properties depending on the photoelectrode thickness using electrochemical impedance analysis. Because of the superior optical properties of PbS QDs, including a wide absorption range as well as thickness optimization, the TiO_2_/PbS QD photoanode exhibited a remarkable photocurrent density of 15.19 mA/cm^2^ at 0.6 V_RHE_ under the condition of 11.9 µm in thickness.

## 2. Materials and Methods

### 2.1. Preparation of Mesoporous TiO_2_ Film

Fluorine-doped tin oxide-coated (FTO) glass substrates (2.2 mm thickness, 8 Ω/sq, Pilkington, Tokyo, Japan) were used as the substrates for the photoelectrode. The FTO substrates were sequentially cleaned using acetone and ethanol in an ultrasonic cleaner (SD-B200H, MUJIGAE, Seoul, Republic of Korea). After cleaning, the substrates were treated with ultraviolet/ozone (UV/ozone cleaner, Yuil Ultra Violet System, YUILUV. Co., Ltd., Incheon, Republic of Korea) for 15 min to remove residual organic materials and moisture. On the cleaned FTO substrates, a solution of 7.5 wt% Ti(IV) bis(ethylacetoacetato)-diiso-propoxide (Aldrich, St Louis, MO, USA) dissolved in n-butanol (Daejung, Siheung, Republic of Korea) was spin-coated, followed by heat treatment at 450 °C for 10 min in a box-type furnace. A semi-transparent TiO_2_ paste (Ti-Nanoxide T/SP, SOLARONIX S.A, Aubonne, Switzerland) was coated on top of the pre-treated FTO substrate using a doctor blading method. The thickness of the TiO_2_ film was controlled by varying the number of layers (1 to 3 layers) of scotch tape attached during the doctor blading. The coated TiO_2_ film was then sintered by gradually increasing the temperature in the box-type furnace: 150 °C for 10 min, 250 °C for 10 min, 400 °C for 10 min, and finally 500 °C for 30 min in air.

### 2.2. Coating of PbS QDs on the Surface of Mesoporous TiO_2_ Film

To coat PbS QDs on the surface of mesoporous TiO_2_ film, the SILAR method was employed. Specifically, PbS QDs were coated by immersing the TiO_2_ film on FTO substrate in a methanol solution of 0.02 M Pb(NO_3_)_2_ (Aldrich) for 60 s, followed by immersion in a solution of 0.02 M Na_2_S (Aldrich) in DI water/methanol (1:1, *v*/*v*). This process was repeated for a total of 4 cycles. Subsequently, a zinc sulfide (ZnS) passivation layer was formed by repeating the SILAR process three times using a solution of 0.05 M Zn(Ac)_2_ (Aldrich) in ethanol (for 50 s), and a solution of 0.05 M Na_2_S in DI water/methanol (1:1, *v*/*v*) (for 50 s).

### 2.3. Characterization

The morphology of the PbS QD-coated TiO_2_ nanoparticles was confirmed by high-resolution transmission electron microscopy (HR-TEM; JEM-2010, JEOL, Tokyo, Japan). The cross-sectional structure of the fabricated photoelectrode was observed using scanning electron microscopy (SEM, S-4700, HITACHI, Tokyo, Japan). Elemental composition was analyzed using energy-dispersive X-ray spectroscopy (EDX) attached to an SEM (Bruker AXS Quantax 4010, HITACHI). The performance of the photoelectrode was measured using a three-electrode system consisting of the photoelectrode, Pt mesh, and Hg/HgO reference electrode in a quartz reactor containing an electrolyte composed of 0.25 M sodium sulfide pentahydrate (Daejung, Siheung, Republic of Korea) and 0.35 M sodium sulfite anhydrous (Daejung, Siheung, Republic of Korea) (pH~13). Impedance data, chronoamperometric curves, and photocurrent density–voltage (*J*–*V*) curves of the photoelectrode were analyzed using a potentiostat (M204, Autolab, Utrecht, The Netherlands). The light incident on the photoelectrode was generated using a 150 W xenon lamp (PEC-L01, Peccell, Yokohama, Japan) equipped with an AM 1.5 G filter. The light intensity was adjusted to 1 sun (100 mW/cm^2^) using a certified silicon reference solar cell. UV-vis transmittance spectra and absorption spectra of the TiO_2_/PbS/ZnS films were achieved using UV-vis spectroscopy (OPTIZEN 2120 UV, Mecasys, Daejeon, Republic of Korea). X-ray photoelectron spectroscopy (XPS, K-alph+, Thermofisher Scientific, E. Grinstead, UK) was used to confirm the surface chemical states of the TiO_2_/PbS/ZnS films. X-ray diffraction (XRD) analyses were carried out using an X-ray diffractometer (SmartLab 9 kW system, Rigaku, Tokyo, Japan). The evolved hydrogen was analyzed by gas chromatography (GC) (Chrozen GC System, YoungIn Chromass, Anyang, Republic of Korea).

## 3. Results and Discussion

**Figure 1:**
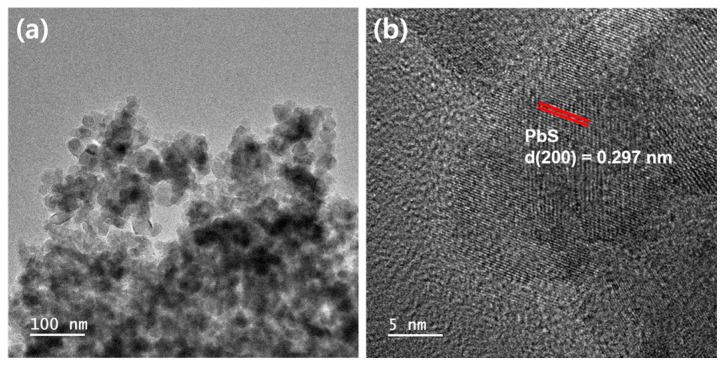
(**a**) HR-TEM image of PbS QD-coated TiO_2_ nanoparticles, and (**b**) high-magnification image of (**a**).

Figure 1a,b show the HR-TEM images of PbS QD-coated TiO_2_ nanoparticles. After coating PbS QDs on the surface of the mesoporous TiO_2_ film via the SILAR method, the film was detached from the substrate for TEM analysis. It was confirmed that TiO_2_ nanoparticles, approximately 20 nm in size, were strongly and randomly bonded to each other through high-temperature annealing (Figure 1a). Particularly, the (200) lattice plane of galena PbS (fringe spacing~0.297 nm) [26] was identified in a highly magnified image (Figure 1b). Figure S1 presents the HR-TEM images displaying the morphology of the bare TiO_2_ nanoparticles. Figure S1a reveals a structural resemblance to that depicted in Figure 1a, suggesting the adsorption of PbS QDs onto the surface of TiO_2_ nanoparticles. The (101) lattice plane of anatase TiO_2_ (fringe spacing~0.353 nm) [27] is identified in Figure S1b. Figure S2 represents the HR-TEM images of the PbS QD-coated TiO_2_ nanoparticles. As shown in Figure S2, the average diameter of the PbS QDs is 4.81 ± 0.82 nm. The observed lattice fringe of 0.297 nm of PbS QDs that corresponds to the (200) plane, indicating the adsorption of PbS QDs over the TiO_2_ nanoparticles.

**Figure 2:**
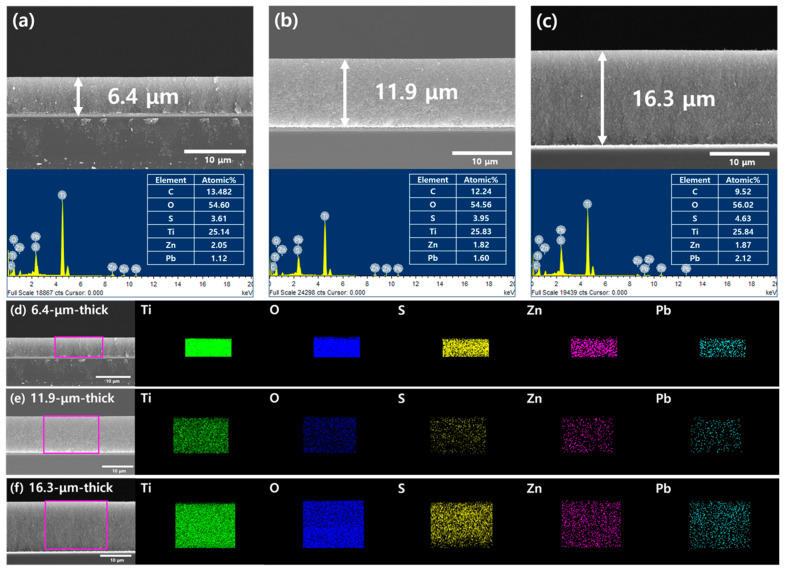
(**a**–**c**) SEM images and EDX spectra for TiO_2_/PbS films according to the thickness. (**d**–**f**) EDX mapping for each element (Ti, O, S, Zn, Pb) of TiO_2_/PbS films.

Figure 2a–c shows SEM images and EDX spectra comparing TiO_2_/PbS/ZnS films with different thicknesses. As observed in the SEM images, the TiO_2_/PbS/ZnS films were coated with varying thicknesses between approximately 6 to 16 µm under each condition. The thickness of these films was controlled by varying the number of layers (one to three layers) of scotch tape attached during the doctor blading of the TiO_2_ paste. The thickness of each film was found to be 6.4 µm (Figure 2a), 11.9 µm (Figure 2b), and 16.3 µm (Figure 2c), respectively. At thicknesses beyond this point, poor adhesion between the TiO_2_ film and the FTO glass substrate led to the formation of cracks, making it difficult to fabricate a stable electrode. The bottom images and tables in Figure 2a–c represent the EDX spectra and the calculated atomic compositions of each electrode, implying the formation of PbS QDs and ZnS passivation layer on the surface of mesoporous TiO_2_ film. From the mapping images in Figure 2d,e, it can be noted that PbS QDs dots and ZnS passivation layer were uniformly and conformally deposited along the cross-section regardless of the thickness of the TiO_2_ film. These results indicate that the SILAR method we employed in this study is suitable for the coating of QDs and the passivation layer onto relatively thick mesoporous electrode surfaces. To investigate whether the coating of QDs influences the thickness of the TiO_2_ electrode, we also measured the thickness of the bare TiO_2_ electrode without QD coating (Figure S3). Depending on the number of layers, the thickness for each layer was determined to be 6.2 µm for a single layer (Figure S3a), 11.3 µm for two layers (Figure S3b), and 16.8 µm for three layers (Figure S3c). These values show only a minor difference within 1 µm compared to the electrode thickness after QD coating, which is considered to be within the expected margin of error associated with the Dr blading method for the TiO_2_ film deposition. Furthermore, the mapping presented in Figure 2d–f indicates that PbS QDs are coated within the pores of the TiO_2_ rather than forming an additional layer on the TiO_2_ film. In conclusion, it can be stated that the coating of QDs does not significantly affect the thickness of the electrode.

**Figure 3:**
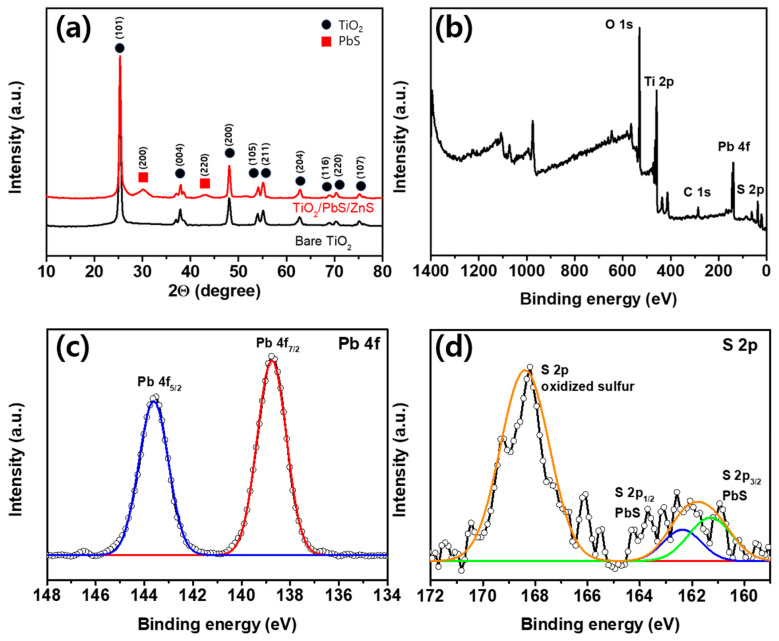
(**a**) XRD spectra of bare TiO_2_ and TiO_2_/PbS/ZnS films. (**b**–**d**) XPS spectra of TiO_2_/PbS film. Survey scan (**b**) and high-resolution scans of Pb 4f (**c**) and S 2p region (**d**) (black lines with circle: measured spectrum, colored lines: fitted curves).

Figure 3a is a graph analyzing XRD patterns to characterize the crystal structure and crystallinity of bare TiO_2_ and TiO_2_/PbS/ZnS films. For the XRD analysis, the 11.9 µm thick film was used. Multiple diffraction peaks observed at 25.3, 37.9, 48.1, 54.0, 55.1, 63.8, 68.9, 70.3 and 74.3 2*θ* degrees in the XRD pattern correspond to the (101), (004), (200), (105), (211), (204), (116), (220) and (107) crystal planes of TiO_2_ with an anatase phase [28,29,30,31,32,33]. Additionally, two diffraction peaks at 30.1 and 43.0 2*θ* degrees for the TiO_2_/PbS/ZnS film represent the (200) and (220) planes of PbS in the galena phase [34,35,36,37,38]. The Gaussian fitting for the (200) plane peak of PbS revealed a peak position at 30.2 2*θ* degrees and a full width at half maximum (FWHM) of approximately 1.75. The average crystallite size can be determined using Scherrer’s equation [39], which is shown in Equation (1):

(1) $$D=\frac{K\lambda }{\beta cos\theta }$$

where *D* represents the average crystallite size, *λ* is the X-ray wavelength (1.5406 Å), *K* is the dimensionless coefficient of particle shape (assumed to be 0.9), *β* is the FWHM in radians, and *θ* is the Bragg angle of diffraction. Based on this equation, the measured average crystallite size for PbS quantum dots deposited on the surface of mesoporous TiO_2_ film was calculated to be approximately 4.71 nm. In our analysis, no impurity peaks, with the exception of TiO_2_ and PbS, were detected, signifying the absence of other crystalline forms within the samples. The absence of the ZnS peak is ascribed to the amorphous nature of ZnS in our case [40]. 

Figure 3b–d present the results of the XPS analysis conducted to verify the chemical states of the TiO_2_/PbS QD film. As seen in the survey scan of Figure 3b, Ti, O, Pb, S, and C are identified in the TiO_2_/PbS QD film. Figure 3c,d represents the high-resolution Pb 4f and S 2p spectra, respectively. The Pb 4f peaks exhibit binding energies (BEs) of 138.8 eV (Pb 4f_7/2_) and 143.6 eV (Pb 4f_5/2_), consistent with values for Pb-S bonds [41]. Additionally, the S 2p peaks have BEs of 161.2 eV (S 2p_3/2_) and 162.3 eV (S 2p_1/2_), also corresponding to values for Pb-S bonds [42]. The additional peaks at higher BEs at 166–171 eV exhibit the presence of oxidized sulfur groups, such as SO_4_^2−^ [43] or SO_3_^2−^ [44], indicating the partial oxidation of the PbS QD surface. This observed outcome is presumed to be a result of the SILAR process conducted in ambient air, which aligns with findings reported in previous studies [45].

**Figure 4:**
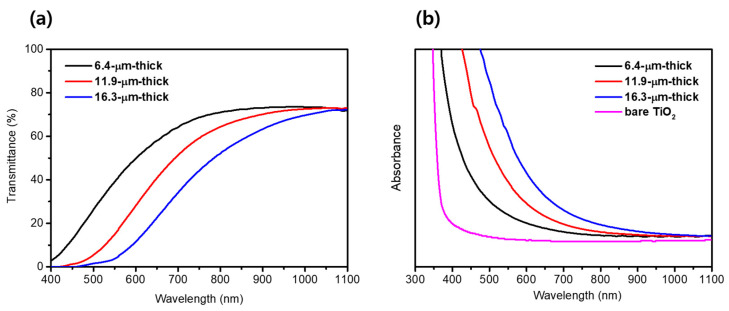
(**a**) Transmittance spectra of TiO_2_ /PbS /ZnS films. (**b**) Absorbance spectra of bare TiO_2_ and TiO_2_/PbS/ZnS films.

Figure 4a,b shows the transmittance spectra and absorption spectra measured using UV-vis spectroscopy to evaluate the optical properties of the TiO_2_/PbS/ZnS films depending on the thicknesses. As shown in Figure 4a, the light transmittance gradually decreases with the increasing thickness of the transparent layer. Conversely, the absorbance of the film increases progressively in the wavelength range of 400 nm to 1100 nm as the thickness of the transparent layer becomes thicker, as shown in Figure 4b. These results arose from the absorption of incident light by the PbS QDs, and as the thickness of the TiO_2_/PbS/ZnS film increased, the loading of PbS QD was also enhanced, leading to a proportional increase in the absorbance. The improvement in absorbance across the entire visible and near-infrared (IR) spectrum is attributed to the narrow band-gap of PbS QDs. According to the previous literature [46], PbS exhibits an optical band-gap of approximately 0.41 eV in its bulk state; however, in the form of QDs with a size of approximately 4.8 nm, it demonstrates an optical band-gap of around 1.00 eV due to the quantum confinement effect. Given that the PbS QDs synthesized through the SILAR method in this study have a size of approximately 4.71 nm, it can be inferred that these QDs possess an optical band-gap slightly larger than 1.00 eV, absorbing photons of up to around 1200 nm wavelengths. This result indicates that as the film thickness increases, the loading of PbS QDs is enhanced, consequently improving the light absorption capability of the TiO_2_/PbS/ZnS film across the entire visible and near-IR spectrum.

**Figure 5:**
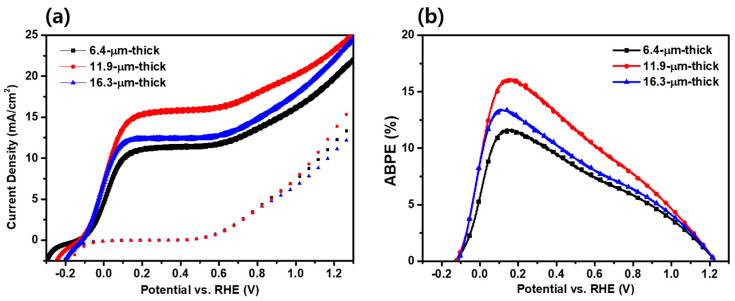
(**a**) *J*–*V* curves and (**b**) ABPE curves of TiO_2_ /PbS /ZnS photoanodes according to the thickness.

Figure 5a shows the *J*–*V* characteristics of the TiO_2_/PbS/ZnS photoanodes depending on the thickness. The performance of each photoelectrode was measured under a 1-sun (100 mW/cm^2^) condition using a three-electrode system within a quartz reactor. The 11.9 µm thick photoanode exhibited the highest photocurrent density, with a value of 15.19 mA/cm^2^ at 0.6 V_RHE_. The other photoanodes showed photocurrent density values of 10.80 mA/cm^2^ (6.4 µm thick) and 11.93 mA/cm^2^ (16.3 µm thick), respectively, under the same condition. Compared to the 6.4 µm thick and 16.3 µm thick photoanodes, the photocurrent density was enhanced by 40.6% and 27.3%, respectively, for the optimized condition (11.9 µm thick), meaning that the photocatalytic activity of QD-decorated TiO_2_ for the sulfite oxidation was greatly affected by the film thickness. The obtained photocurrent density of 15.19 mA/cm^2^ from this study indicates a promising outcome for QD-decorated photoelectrodes when compared to previous reports as listed in Table S1 [22,47,48,49,50,51,52,53]. 

Figure 5b shows the applied bias photon-to-current efficiency (ABPE) curves calculated using the following Equation (2) [54,55]:

(2) $$\left(\frac{J_{ph}\times \left(1.23-V_{app}\right)}{P_{in}}\right)\times 100\%$$

where *J*_ph_ represents the measured current density, *V*_app_ is the applied external potential versus RHE and *P*_in_ is the power density of the incident light. The ABPE results aligned with the observed patterns in photocurrent density. Specifically, the 11.9 µm thick photoanode displayed the highest ABPE, reaching 16.17% at 0.153 V. In comparison, the 6.4 µm thick photoanode exhibited an ABPE of 11.70% at 0.143 V, while the 16.3 µm thick photoanode demonstrated an ABPE of 13.43% at 0.109 V. 

**Figure 6:**
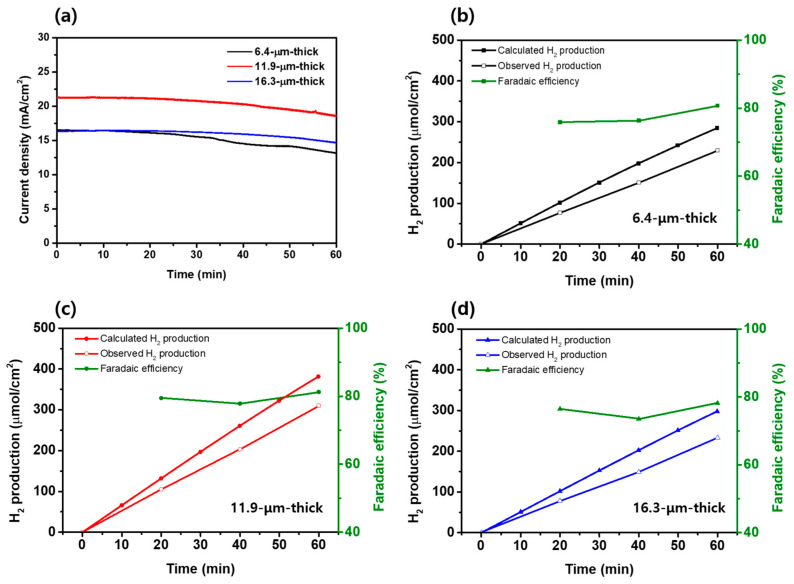
(**a**) Chronoamperometric curves (at 0.6 V_RHE_) of TiO_2_/PbS/ZnS photoanodes. (**b**–**d**) Hydrogen production over time measured at 0.6 V_RHE_ for 1 h, calculated theoretical hydrogen yield, and calculated faradaic efficiency for TiO_2_/PbS/ZnS photoanodes according to the thickness.

To assess photostability, chronoamperometry was conducted at 0.6 V_RHE_ for one hour, as shown in Figure 6a. The reduction in photocurrent density were observed as follows: 20.45% for the 6.4 µm thick photoanode, and 11.19% and 11.85% for the 11.9 µm thick and the 16.3 µm thick photoanode, respectively. As discussed above, as the thickness of the photoanode increases, the loading of PbS QDs is improved, leading to the enhanced absorbance and PEC performance of a photoanode. However, beyond a certain threshold, it is speculated that unfavorable factors such as increased electron recombination might lead to decreased performance [19,20]. Regardless of the thickness, it was observed that the photocurrent density decreased after 1 h in all three types of photoelectrodes. This is believed to be due to the decreased stability resulting from the facile oxidation of metal chalcogenides when used as electrodes in PEC hydrogen production [56,57]. This insufficient stability observed under illumination can be improved by applying effective overlayers for the passivation [10,58].

As represented in Figure 6b–d, the theoretical hydrogen generation was evaluated based on the chronoamperometry curves in Figure 6a, from Equation (3) [59]:

(3) $${Moles\;of\;H}_{2}=\frac{1}{2}\frac{\int_{0}^{t}Idt}{F}$$

where *F* represents the Faraday constant, *I* is the measured photocurrent, and *t* is the measured time. The theoretically calculated hydrogen productions for each photoanode over the one hour are as follows: 284 µmol/cm^2^ (6.4 µm thick; Figure 6b), 382 µmol/cm^2^ (11.9 µm thick; Figure 6c), and 298 µmol/cm^2^ (16.3 µm thick; Figure 6d). Compared to the 6.4 µm thick and 16.3 µm thick photoanode, the hydrogen production was enhanced by 34.5%, 28.2%, respectively, for the optimized thickness condition (11.9 µm thick). The actual hydrogen production was measured at 0.6 V_RHE_ for 1 h, as shown in Figure 6b–d. For each photoanode, the faradaic efficiency was retained between 73~81%. The 6.4 μm thick photoanode produced 230 μmol/cm^2^ over a period of 1 h (Figure 6b), the 11.9 μm thick photoanode produced 310 μmol/cm^2^ (Figure 6c), and the 16.3 μm thick photoanode produced 233 μmol/cm^2^ (Figure 6d), respectively. These results are matched well with the *J*–*V* curves for each photoanode, and indicate that the optimization of the photoanode thickness not only maximized the photocatalytic activity for the sulfite oxidation but also led to the actual maximum hydrogen production.

**Figure 7:**
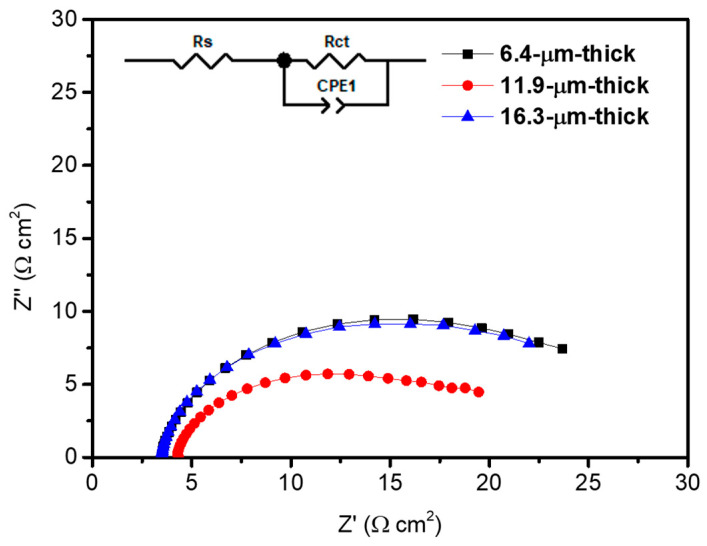
Electrochemical impedance spectra of TiO_2_ /PbS /ZnS photoanodes according to the thickness under the 1-sun condition (100 mW/cm^2^) at 0.6 V_RHE_. The inset represents the equivalent circuit used for the fitting of impedance spectra.

In addition, to thoroughly examine the influence of photoanode thickness on the charge transfer property, we conducted impedance analysis, as shown in Figure 7. The impedance spectra were obtained under the 1-sun condition (100 mW/cm^2^) at 0.6 V_RHE_, with a frequency range from 0.1 Hz to 100 kHz, and a sinusoidal perturbation of 10 mV. Using the equivalent circuit shown in Figure 7, we performed fitting in the Z-view program. The employed equivalent circuit consists of *R*_S_ (solution resistance), *R*_ct_ (charge transfer resistance), and CPE1 (constant phase element). The CPE represents the interfacial capacitance, which generally replaces a capacitor in the equivalent circuit for electrodes with high roughness [42]. The CPE parameter includes two parts: CPE-*T*, which represents the value of the capacitance, and CPE-*P*, known as the magnitude of the compression from an ideal semicircle in the Nyquist plot [60]. The *R*_ct_ and CPE1 are related to the charge transfer characteristics at the interface of photoanode/redox electrolyte [10,61]. The values of each parameter corresponding to the thickness of the photoanode are listed in Table 1, and the value of the CPE parameters are listed in Table S2. The *R*_S_ values were similar among the three photoanodes; however, the *R*_ct_ value significantly decreased for the 11.9 µm thick photoanode. Compared to the 6.4 µm thick and 16.3 µm thick photoanode, it decreased by 37.1% and 30.0%, respectively, for the 11.9 µm thick photoanode, meaning that the charge transfer process occurs most efficiently for the 11.9 µm thick condition. This result is attributed to the improved light absorption capability compared to the 6.4 µm thick photoanode, and more favorable mass transfer of the electrolyte compared to the 16.3 µm thick photoanode.

## 4. Conclusions

In summary, our research underscores the pivotal role of optimizing the structure of PbS QD-decorated mesoporous TiO_2_ photoanodes to enhance the PEC hydrogen production performances. Through systematic investigation, we observed a clear correlation between the thickness of the photoanodes and their performance. Increasing the thickness of the photoanodes, within certain limits, resulted in enhanced light absorption capabilities across the visible and near-infrared spectrum. However, our findings demonstrate that the 11.9 µm thick photoanode yielded the most favorable balance, exhibiting an optimal photocurrent density of 15.19 mA/cm^2^, compared to the 6.4 µm thick and 16.3 µm thick counterparts. This optimal performance was attributed to the trade-off between its increased light absorption capacity and efficient electrolyte mass transfer, which was confirmed using the electrochemical impedance analysis by revealing the lowest charge transfer resistance in the 11.9 µm thick photoanode. The insights gained from this study will pave the way for the development of high-performance PEC hydrogen production devices.

## Figures and Tables

**Table 1:** Summary of *J*–*V* characteristics and electrochemical impedance parameters for TiO_2_/PbS/ZnS photoanodes according to the thickness.

	Current Density (mA/cm^2^)	*R*_S_ (Ω cm^2^)	Error (%)	*R*_ct_ (Ω cm^2^)	Error (%)
6.4 µm thick	10.80	3.39	0.45	23.77	0.61
11.9 µm thick	15.19	4.17	0.57	14.95	0.84
16.3 µm thick	11.93	3.43	0.44	21.35	0.74

All data were measured at 0.6 V_RHE_.

## Data Availability

All data are contained within the article.

## Supplementary Materials

The following supporting information can be downloaded at: https://www.mdpi.com/article/10.3390/ma17010225/s1, Figure S1: (a) HR-TEM image and (b) lattice fringe of bare TiO_2_ nanoparticles; Figure S2: HR-TEM images of PbS QDs on the surface of TiO_2_ nanoparticles with lattice fringe; Figure S3: (a–c) Cross-sectional SEM images of bare TiO_2_ films according to the thickness; Table S1: Previously reported performances of QD-decorated photoelectrodes prepared by SILAR process for PEC hydrogen production; Table S2: Summary of fitting results of CPE parameters from EIS.
