# Supplementary material for: Investigating the Influence of PbS Quantum Dot-Decorated TiO2 Photoanode Thickness on Photoelectrochemical Hydrogen Production Performance
Source: Materials (Basel). 2023 Dec 31;17(1):225. doi: 10.3390/ma17010225 (PMC10779549; doi:10.3390/ma17010225)
Supplement: Supplementary file 1 [file materials-17-00225-s001.zip › materials-2751943-supplementary.pdf]

materials

Supplementary Materials

Table S1. Previously reported performances of QD-decorated photoelectrodes prepared by SILAR process for PEC hydrogen production.

Photoelectrode	Electrolyte conditions	Photocurrent density (mA/cm^2)	Reference
ZnO nanowire with CdSe/CdS QDs	0.25 M Na_2S with 0.35 M Na_2SO_3 (pH = 12.5)	–	[47]
TiO_2 nanotube with Bi_2S_3 QDs	0.1 M Na_2S $9H_2O$ with 0.1 M Na_2SO_3 (pH = 12.5)	1.27 (at 0.93 v vs. RHE)	[48]
CdS/TiO_2 nanorod with CdS QDs	0.25 M Na_2S with 0.35 M Na_2SO_3 (pH = 13.13)	2.03 (at 0.8 V vs. RHE)	[49]
TiO_2 hyperbranched nano-trees with CdS QDs	0.25 M Na_2S $9H_2O$ with 0.35 M Na_2SO_3 (pH = 12.4)	4.06 (at 0 V vs. RHE)	[50]
3D sea urchin like TiO_2 with CdS QDs	0.25 M Na_2S with 0.35 M Na_2SO_3 (pH = 12.5)	6.37 (at 0.93 V vs. RHE)	[51]
CdS/ $BaSnO_3$ nanowire with CdS QDs	0.25 M Na_2S with 0.35 M Na_2SO_3 (pH = 12.8)	5.80 (at 1.00 V vs. RHE)	[52]
ZnO nanosheets with CdS QDs	0.1 M Na_2SO_4	–	[53]
PbS/Mn-doped CdS QDs	0.25 M Na_2S with 0.35 M Na_2SO_3 (pH = 12.5)	22.17 (at 0.6 V vs. RHE)	[22]
PbS QD decorated mesoporous TiO_2	0.25 M Na_2S $5H_2O$ with 0.35 M Na_2SO_3 (pH = 13)	15.19 (at 0.6 V vs. RHE)	This work

Table S2. Summary of fitting results of CPE parameters from EIS.

Photoelectrodes	CPE			
	T ($x10^{-5}$)	Error (%)	P	Error (%)
6.4-μm-thick	4.22	0.48	0.87	0.47
11.9-μm-thick	5.84	0.68	0.85	0.59
16.3-μm-thick	2.59	0.45	0.90	0.37

All data were measured at 0.6 V_{RHE}.

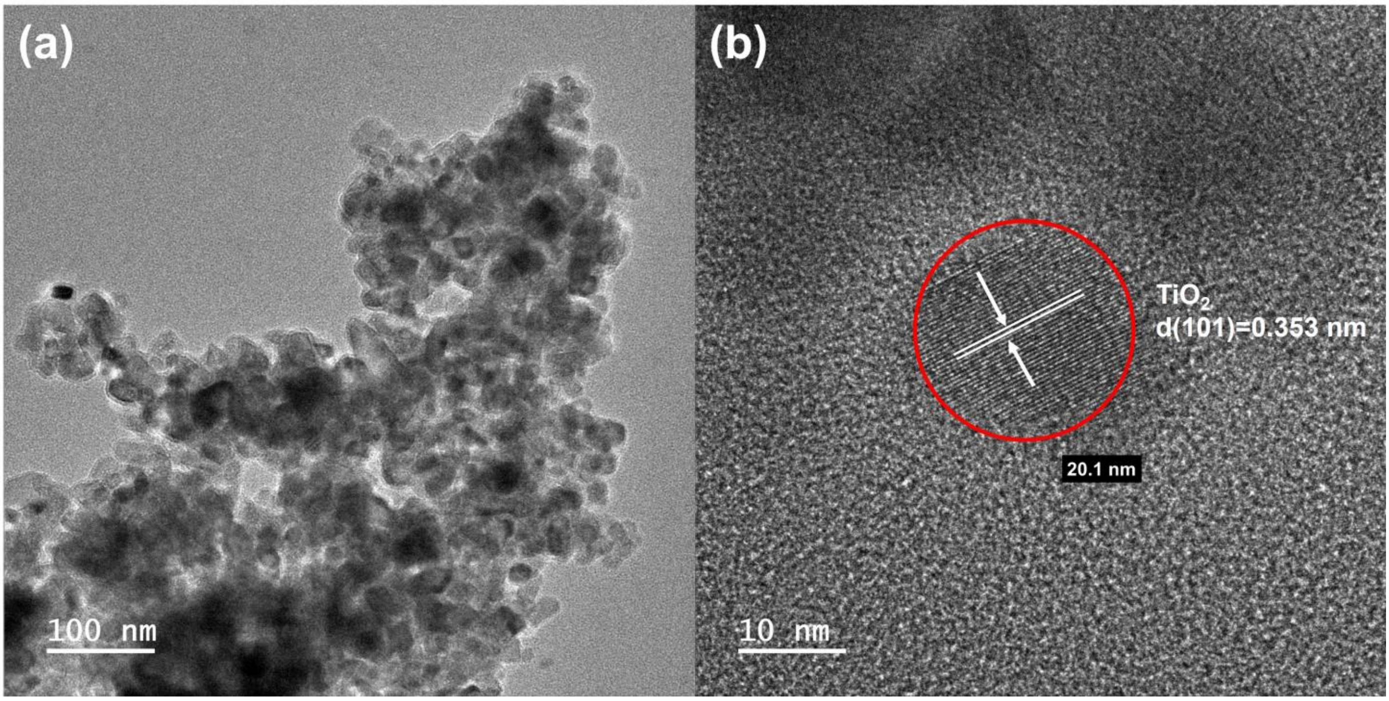

Figure S1. (a) HR-TEM image and (b) lattice fringe of bare TiO_2 nanoparticles.

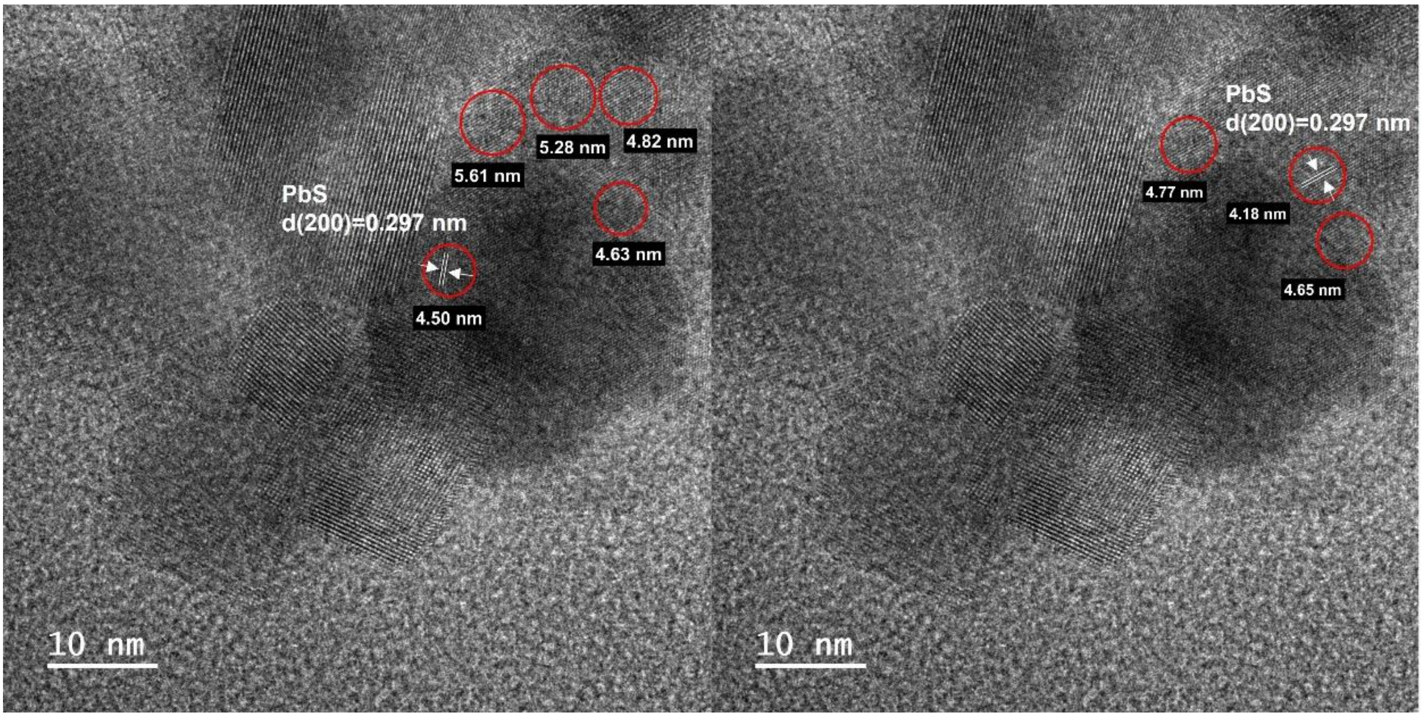

Figure S2. HR-TEM images of PbS QDs on the surface of TiO_2 nanoparticles with lattice fringe.

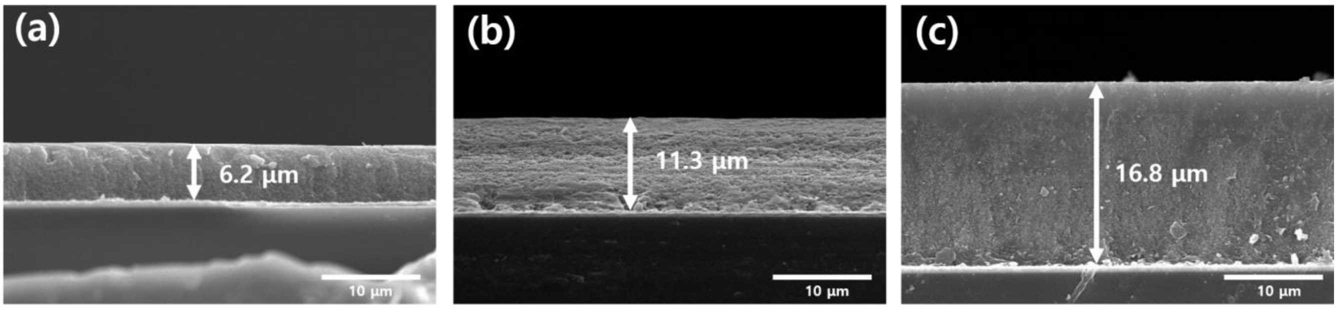

Figure S3. (a-c) Cross-sectional SEM images of bare TiO_2 films according to the thickness.
